# Wesselsbron Virus‐Induced Hepatitis in Ewes and Lambs Unraveled Through Machine Learning‐Driven Digital Histopathology

**DOI:** 10.1155/tbed/7912840

**Published:** 2026-02-11

**Authors:** Llorenç Grau-Roma, Simone de Brot, Marta Zimoch, Loane Clerc, Noelle Donzé, Matthias Liniger, Francisco Brito, Adrián Herrera, Aurélie Godel, Artur Summerfield, Charaf Benarafa, Obdulio García-Nicolás

**Affiliations:** ^1^ COMPATH, Institute of Animal Pathology, Department of Infectious Diseases and Pathobiology, Vetsuisse Faculty, University of Bern, Länggassstrasse 122, Bern, 3012, Switzerland, unibe.ch; ^2^ Multidisciplinary Center for Infectious Diseases, University of Bern, Bern, Switzerland, unibe.ch; ^3^ Department of Infectious Diseases and Pathobiology, Vetsuisse Faculty, University of Bern, Bern, Switzerland, unibe.ch; ^4^ Institute of Virology and Immunology (IVI), Sensemattstrasse 293, Mittelhäusern, 3147, Switzerland; ^5^ Graduate School for Cellular and Biomedical Sciences (GCB), University of Bern, Bern, Switzerland, unibe.ch

**Keywords:** flavivirus, hepatitis, immunohistochemistry, machine learning, quantification, ruminants, zoonosis

## Abstract

Wesselsbron virus (WSLV) disease is an important neglected cause of hepatitis in ruminants with potential for zoonotic transmission, yet its histological lesions have been scarcely studied. We performed a thorough machine learning‐driven, pathologist‐led digital histopathological assessment of WSLV–induced hepatitis in ewes and lambs infected with clade I (rSA999) (*n* = 6) or clade II (SAH117) (*n* = 8) strains and a mock group (*n* = 6). The analysis was performed on immunohistochemical (IHC) staining for T cells (CD3), B cells (PAX5), histiocytes (Iba1), the WSLV nonstructural protein 1 (NS1), and a double stain for arginase 1 and Ki67 to assess the hepatocyte proliferation index (PI). WSLV–infected animals exhibited significantly higher lymphohistiocytic infiltration and higher hepatocyte PI compared to the mock group. The T cell density was 10 folds higher than the B cell density and was more pronounced in the rSA999 group than in the SAH177 group. Digitally quantified parameters positively correlated with WSLV reverse transcription quantitative PCR (RT‐qPCR) results and hepatic injury markers (aspartate transferase [AST], bilirubin, and adenosine deaminase [ADA]), indicating that digital histopathology reliably detects liver damage and disease severity. Among the parameters assessed, the positive correlation between the density of Iba1^+^ staining and the WSLV viral load in the liver was the strongest, underscoring the prominent involvement of histiocytes in WSLV–induced hepatitis. This study demonstrates the value of digital histopathological analysis in viral‐induced hepatitis using formalin‐fixed paraffin‐embedded (FFPE) tissue, leveraging whole‐slide imaging and deep learning (DL) to objectively characterize key hepatic alterations caused by the viral infection.

## 1. Introduction

*Orthoflavivirus wesselsbronense*, commonly known as Wesselsbron virus (WSLV), is a zoonotic, neglected, and emerging mosquito‐borne orthoflavivirus that is phylogenetically related to the yellow fever virus. While it primarily affects domestic and wild ruminants in sub‐Saharan Africa [1–3], WSLV can also infect humans, usually causing self‐limiting febrile illness and rarely leading to neurological complications [4]. Very recently, WSLV has been suggested to be the cause of an outbreak of severe disease with 70% of mortality in dromedary camels calves in Ethiopia [5]. Although reports of WSLV outbreaks are restricted to South Africa, recent WSLV detection in mosquitoes, rats, and humans from other African countries suggest a wider distribution [1, 2]. Two genetic clades of WSLV circulate in sub‐Saharan Africa: the more recently emerging and currently predominant clade I WSLV strains and the clade II strains [4], both belonging to a single serotype [6]. Only very few studies have shed light on the pathogenicity of the more recently circulating clade I WSLV strains [6].

WSLV infection in adult sheep is often subclinical, leading to abortion and stillbirth in pregnant ewes. Severe disease is observed in neonates, causing significant economic losses due to high perinatal mortality [3, 7]. The main target organ and cell target of WSLV are the liver and hepatocytes, respectively, making hepatic pathology a crucial aspect of disease characterization. WSLV–induced hepatic lesions are characterized by hepatocellular necrosis scattered throughout the parenchyma, a predominantly lymphocytic infiltrate, and Kupffer cell (Kc) hyperplasia [8, 9]. In line with the typical clinical presentation, prominent hepatocellular necrosis is a feature that may be observed in newborn lambs but is rarely present in adult animals [9, 10]. Moreover, histological evidence of secondary regeneration of hepatic parenchyma has been suggested based on the nonquantitative observations of an increased number of mitotic figures and enlarged nuclei in hepatocytes from WSLV–infected ovine livers [9].

We have previously demonstrated a new mode of vector‐independent transmission of WSLV from lactating ewes to their lambs [6]. This study demonstrated vector‐free horizontal transmission of WSLV to suckling lambs, which were housed with ewes intravenously inoculated with WSLV. The blood biochemical analysis suggested a more severe hepatic damage in animals infected with a recombinant strain from the clade I (rSA999) than in animals infected with a field‐derived strain from the clade II (SAH177), which has been maintained through serial laboratory passages. Nevertheless, the semiquantitative histopathological assessment did not allow demonstration of significant differences between groups.

Digital pathology, encompassing whole‐slide images (WSIs), machine learning‐driven image analysis, and quantitative histopathology, allows for high‐throughput, standardized, and reproducible histological assessment, offering new opportunities to enhance the understanding of infectious diseases [11]. Nevertheless, compared to its already extensive use in cancer research, its application in infectious disease research is still limited [12, 13].

This study presents an in‐depth, fully quantitative and automated digital histopathological analysis in WSI of formalin‐fixed paraffin‐embedded (FFPE) liver samples from our prior experiment [6]. Specifically, we quantitatively assessed immunohistochemical (IHC) markers for T and B lymphocytes, histiocytes/macrophages, WSLV antigen, and hepatocellular proliferation. This study aimed to evaluate the ability of the digital quantification of IHC markers to distinguish WSLV–infected from noninfected animals as well as rSA999 from SAH177–infected groups and to assess their correlation with previously acquired data of WSLV viral load in liver and of clinical chemistry markers of hepatic damage.

## 2. Materials and Methods

### 2.1. Ethics Statement

The animal experimentation on sheep (Ovis orientalis aries), Skudde breed, was conducted in the Biosafety Level‐3 Agriculture (BSL3Ag) biocontainment stables, insect‐free facilities of the Institute of Virology and Immunology (IVI, Mittelhäusern) in strict compliance with the Swiss animal protection law (Article 18 Animal Welfare Act (SR 455), Article 141 Animal Welfare Ordinance (SR 455.1), and Article 30 Animal Experimentation Ordinance (SR 455.163)), following good animal practice as defined by European regulations, and approved by the Cantonal ethical committee for animal experiments under the license number BE69/2022.

### 2.2. Liver Samples

Liver samples (*n* = 20) from a previous experimental infection were used for this study [6], including ewes (*n* = 13) and lambs (*n* = 7). Key biological variables did not differ between groups. Shortly, in the previous study [6], lactating ewes were intravenously inoculated with 10^5^ TCID_50_ of either clade I (rSA999) (*n* = 5), clade II (SAH177) (*n* = 5), or mock‐inoculated with C6/36 cell culture supernatant (*n* = 3). Each group of ewes with their respective noninoculated lambs (*n* = 14) was housed isolated from each other in BSL3Ag biocontainment stables. WSLV replication and viremia occurred in all WSLV–inoculated ewes and in five lambs (two and three from rSA999 and SAH177 groups, respectively). Expectedly, WSLV was not detected in any animal of the mock group. Euthanasia and postmortem examination of all animals was performed at 12 days postinfection (dpi), except for one lamb from the group rSA999, which died during the night at 11 dpi and was necropsied the following morning. FFPE liver samples from the 14 WSLV–infected animals (rSA999, *n* = 6, 5 ewes and 1 lamb; SAH177, *n* = 8, 5 ewes and 3 lambs) as well as from the mock group (*n* = 6, 3 ewes and 3 lambs) were included in this study. The lamb that died on its own was excluded from the study due to autolysis, which could affect the IHC staining.

### 2.3. Histopathology

FFPE liver samples were cut at 4 μm of thickness and stained with hematoxylin and eosin (H&E) for histological examination. Hepatic lesions were semiquantitatively scored by a board‐certified pathologist (LGR), as previously described [6], from 0 to 3 as follows: 0: none; 1: mild; 2: moderate; and 3: severe.

### 2.4. Immunohistochemistry

Serial sections of the FFPE liver samples were cut at 2 µm of thickness, mounted on positively charged slides, and dried for 30 min at 60 °C and subsequently dewaxed, pretreated, and stained on an Immunostainer Leica Bond RX (Leica Biosystems) as follows. Section slides were dewaxed (Bond Dewax solution; Leica Biosystems) and subjected to a heat‐induced epitope retrieval step, using the temperature condition buffers listed in Table 1, which also summarizes the antibodies and staining conditions used. In the case of IHC for WSLV, a protein block (FBS 1%) was used to reduce nonspecific binding of the primary antibody. The slides were then incubated with the corresponding primary antibodies for the single staining of ionized calcium‐binding adapter molecule‐1 (Iba1), CD3, paired box 5 protein (PAX5), and WSLV NS1, or for the sequential double staining of proliferation marker protein Ki67 (Ki67) and arginase 1 (Arg1) using the conditions detailed in Table 1. Antibodies were diluted manually in Bond Primary Antibody diluent (Leica Biosystems, #AR9352) and added on BOND RX separately. All further steps were performed using reagents of the Bond Polymer Refine Detection Kits (Leica Biosystems), using either an antirabbit poly‐horseradish peroxidase (HRP)–IgG linked reagent (15 min) or a secondary Rabbit antimouse IgG reagent (15 min) to localize primary rabbit and mouse antibodies, respectively. For the single IHC and for Ki67, visualization was achieved using 3,3^′^‐diaminobenzidine tetrahydrochloride hydrate (DAB) (10 min) for brown chromogen (BOND Polymer Refine Detection kit, DS9800). Finally, all slides were counterstained with hematoxylin and mounted with Eukitt (Sigma–Aldrich).

**Table 1:** List of antibodies and conditions used for immunohistochemistry.

Antibody	Type of antibody (clone)	Dilution, incubation time, and temperature	Antigen retrieval	Source	Catalog number
CD3	Mouse mAb (LN10)	1:400, 30 min, RT	Tris EDTA^a^ 20 min at 95 °C	Novocastra	NCL‐LC‐D3‐565
Iba1	Rabbit mAb (EPR16588)	1:10,000, 30 min, RT	Tris EDTA^a^ 30 min at 95 °C	Abcam	ab178846
Pax5	Rabbit mAb (RBT‐Pax)	1:100, 30 min, RT	Tris EDTA^a^ 30 min at 95 °C	BioSB	BSB5864
Ki67	Mouse mAb (MIB‐1)	1:200, 30 min, RT	Tris EDTA^a^ 30 min at 95 °C	DAKO Agilent	M7240
Arg1 (H‐52)	Rabbit pAb	1:50, 30 min, RT	Citrate^b^ 30 min at 100 °C	Santa Cruz	sc‐20150
WSLV NS1	Mouse mAb^c^	1:100, 60 min, RT	Citrate 30 min with protein block (FBS 1%) at 100 °C	Medix Biochemica	HM1022

*Note:* WSLV NS1, Wesselsbron virus nonstructural protein 1.

Abbreviations: Arg1, arginase 1; FBS, fetal bovine serum; mAB, monoclonal antibody; pAb, polyclonal antibody; RT, room temperature.

^a^Bond Epitope Retrieval 1, pH 6 (Leica Biosystems).

^b^Bond Epitope Retrieval 2, pH 9 (Leica Biosystems).

^c^No clone number available.

For the double staining, a first cycle of immunolabeling using Ki67 was performed as described above without hematoxylin counterstain. Subsequently, Arg1 was automatically stained in the second cycle followed by the detection with BOND Polymer Refine RED Detection Kit (DS9390), including the hematoxylin counterstain. Finally, slides were mounted with Aquatex (Sigma–Aldrich).

Ovine tissues known to be positive for all the assessed IHC markers were included on each run as positive controls. Additionally, negative controls were performed replacing the primary antibody by antibody diluent.

### 2.5. Digital Analysis

The Visiopharm software (Version 2024.07, Hørsholm, Denmark) was used to perform quantitative digital histological analysis. The IHC glass slides were digitized with NanoZoomer S360 digital slide scanner (Hamamatsu Photonics K.K., Hamamatsu City, Japan) for WSI analysis. WSI images were produced at a resolution of 40×, corresponding to 0.23 µm/pixel. The generated files were in the “.ndpi” format. Available liver tissue was defined as “region of interest” (ROI), which was fully assessed using different application protocol packages (APPs) as described below. In all analytic steps, manual revision of the generated tissue labels and corrections was performed in each slide before generating the results. Manual corrections were minor and consisted mostly of excluding tissue artifacts and unspecific staining.

1. Tissue detection. The pixel classifier decision forest at magnification 0.5× was used to detect tissue present on the slide and to outline the generated tissue label as ROI. The area with the liver sample was first manually marked, and subsequently, the APP allowed distinction between background and tissue.
2. CD3–positive cell detection. A deep learning (DL) (U‐Net) classification at magnification 20× and 100,000 iterations was used to identify individual positive stained cells. The total and relative (cell count per mm^2^ liver tissue) CD3–positive cell counts were calculated.
3. PAX5–positive cell detection. A predesigned DL–based APP available in the Visiopharm APP Center (https://visiopharm.com/app-center/) (APP #10170), which had been designed for IHC nuclei detection, was used after adjusting the threshold for hematoxylin counterstain combined with DAB (HDAB‐DAB) positivity. The total and relative (cell count per mm^2^ liver tissue) PAX5–positive cell counts were calculated.
4. Iba1–positive tissue detection. A threshold classification based on the feature HDAB‐DAB was used to detect positive Iba1 staining. The total (tissue area in mm^2^) and relative (% of positivity in relation to the total ROI of each examined liver section) for positive Iba1 tissue area were calculated.
5. Ki67–positive hepatocyte detection. A DL (U‐Net) classification at magnification 20× and 100,000 iterations was used to identify and distinguish the nuclei of hepatocytes (arginase 1 positive) from nuclei from the remaining cells (arginase 1 negative, i.e., leukocytes, vessels, fibroblasts, and bile ducts). In a postprocessing step, only the hepatocellular nuclei were further evaluated for Ki67 expression. Ki67 positivity was defined based on the level of the feature FastRed_DAB‐DAB. The proliferation index (PI) of hepatocytes was calculated as: (Ki67–positive hepatocyte nuclei/total hepatocyte nuclei) × 100.
6. WSLV (NS1)–positive tissue detection. First, a DL (U‐Net) classification at 20× magnification and 100,000 iterations was used to detect and exclude blood vessels with nonspecific WSLV staining. Next, a threshold classification based on the feature HDAB‐DAB was used to detect WSLV positivity. The total (tissue area in mm^2^) and relative (% of staining in relation to the total ROI of each examined liver section) WSLV–positive tissue area were calculated.

### 2.6. Reverse Transcription Quantitative PCR (RT‐qPCR) for Viral RNA and Serum Hepatic Markers

RT‐qPCR on liver samples (copies/g) and serum hepatic makers including aspartate transferase (AST) (IU/L), bile acids (µm), bilirubin (g/dL), and adenosine deaminase (ADA) (IU/L) had already been performed and described [6]. Here, these data were used to perform correlation analysis with the digitally assessed parameters.

### 2.7. Statistical Analysis

GraphPad Prism 10 Software (GraphPad Software, Inc., La Jolla, San Diego, CA, USA) was used for data analysis and generation of the violin plots. Firstly, data distribution normality was assessed by the Shapiro–Wilk test. Thereafter, unpaired, two tailed *t*‐tests were used for two group comparisons, specifically *t*‐test or Mann–Whitney *U* test for normally and non‐normally distributed variables, respectively. The group comparisons were (1) WSLV, Mock; (2) Ewes, Lambs; (3) rSA999, SAH177, mock; and (4) rSA999 ewes, SAH177 ewes, mock ewes. Results were presented as mean values with standard deviation and as median and interquartile range for parametric and nonparametric data, respectively. A *p*  < 0.05 was considered statistically significant, which was indicated in the violin plots by specific *p*‐values. R software (R Foundation for Statistical Computing, Vienna, Austria) package was used to perform the correlation Spearman’s Rho (ρ) analysis and to generate the correlation network plot. The correlation analysis was performed with Benjamini–Hochberg false discovery rate (FDR) correction for multiple testing. Results were reported as FDR–adjusted *q*‐values. An FDR–adjusted *q*‐value < 0.05 was considered statistically significant. In the correlation heatmap, significance is indicated by asterisks ( ^∗^ *q*< 0.05,  ^∗∗^ *q* < 0.01,  ^∗∗∗^ *q* < 0.001, and  ^∗∗∗^ *q* < 0.0001), and only correlations with *R^2^* > 0.5 were considered biologically relevant.

## 3. Results

### 3.1. Liver Pathology

As reported initially [6], the only macroscopical lesions noted in the liver were the presence of few small multifocal white to beige round to linear lesions compatible with *Dicrocoelium dendriticum* (two from rSA999–infected group, two from the SAH177–infected group, and 1 from the mock group), consisting of white, small multifocal areas of 1 mm x1 mm × up to 5 mm in length, with occasional presence of flukes in the liver parenchyma and/or in the gallbladder.

Histologically, multifocal, random necrotizing hepatitis was observed in 10 animals, including four animals (three ewes and one lamb) from the rSA999 group and six animals (three ewes and three lambs) from the SAH177 group. These lesions were mild in all animals except for the lamb from the rSA999 group and one ewe from the SAH177 group, where the lesions were moderate. The necrotizing hepatitis was characterized by individual or small clusters of hypereosinophilic rounded‐off hepatocytes often resembling Councilman bodies, accompanied by a predominantly lymphocytic and histiocytic infiltrate. No lesions of necrotizing hepatitis were observed in the controls (*n* = 3 ewes and *n* = 3 lambs). In addition, hyperplastic lymphocytic cholangitis typical of *Dicrocoelium dendriticum* infestation was observed in 10 out of the 13 ewes (five from the rSA999 group, three from the SAH177 group, and two from the control group).

### 3.2. CD3

Different leukocyte subsets are recruited to the liver during hepatic viral infections to help control the viral replication. T lymphocytes were identified as CD3^+^ cells by immunohistochemistry staining [14]. T cells were predominantly individually scattered within the hepatic parenchyma and in the portal tracts, multifocally forming small aggregates (Figure 1A). Independently of the viral strain, when comparing infected and noninfected animals, CD3 density was higher in the WSLV–infected animals (*p* = 0.0386; Figure 1A,B). In general, CD3^+^ cell density was higher in the liver of rSA999–infected animals compared to the mock group (*p* = 0.0152, Figure 1B). When comparing CD3^+^ cell density only in ewes, T cell density was significantly higher for rSA999–infected ewes compared to the SAH177–infected ones (*p* = 0.0067) or the mock group (*p* = 0.0188).

Figure 1Quantitative digital analysis of CD3 immunohistochemistry. (A) Depicts the histological liver appearance from three representative lambs: one infected with WSLV strain rSA999 (top), one with WSLV strain SAH177 (middle), and one mock (bottom). Images on the left are unlabeled, whereas corresponding images on the right include orange digital labels outlining CD3–positive cells. Scattered CD3–positive cells are present within the liver parenchyma (white arrows) and the portal tracts (black arrows), with formation of small cellular aggregates in the parenchyma of WSLV–infected lambs (arrowhead). Scale bar: 90 µm. Inset: Higher magnification of the corresponding image showing in more detail a small aggregate of CD3–positive cells. Scale bar: 25 µm. (B) CD3–positive cell density. Results are presented as the number of CD3–positive cells/mm^2^ of liver parenchyma. Distinct symbols are used to represent different animals within each group as depicted by Zimoch et al. [6]; lambs have the same symbol as their mother; twin lambs from the SAH177 group are distinguished by a dotted symbol.

**Figure:**
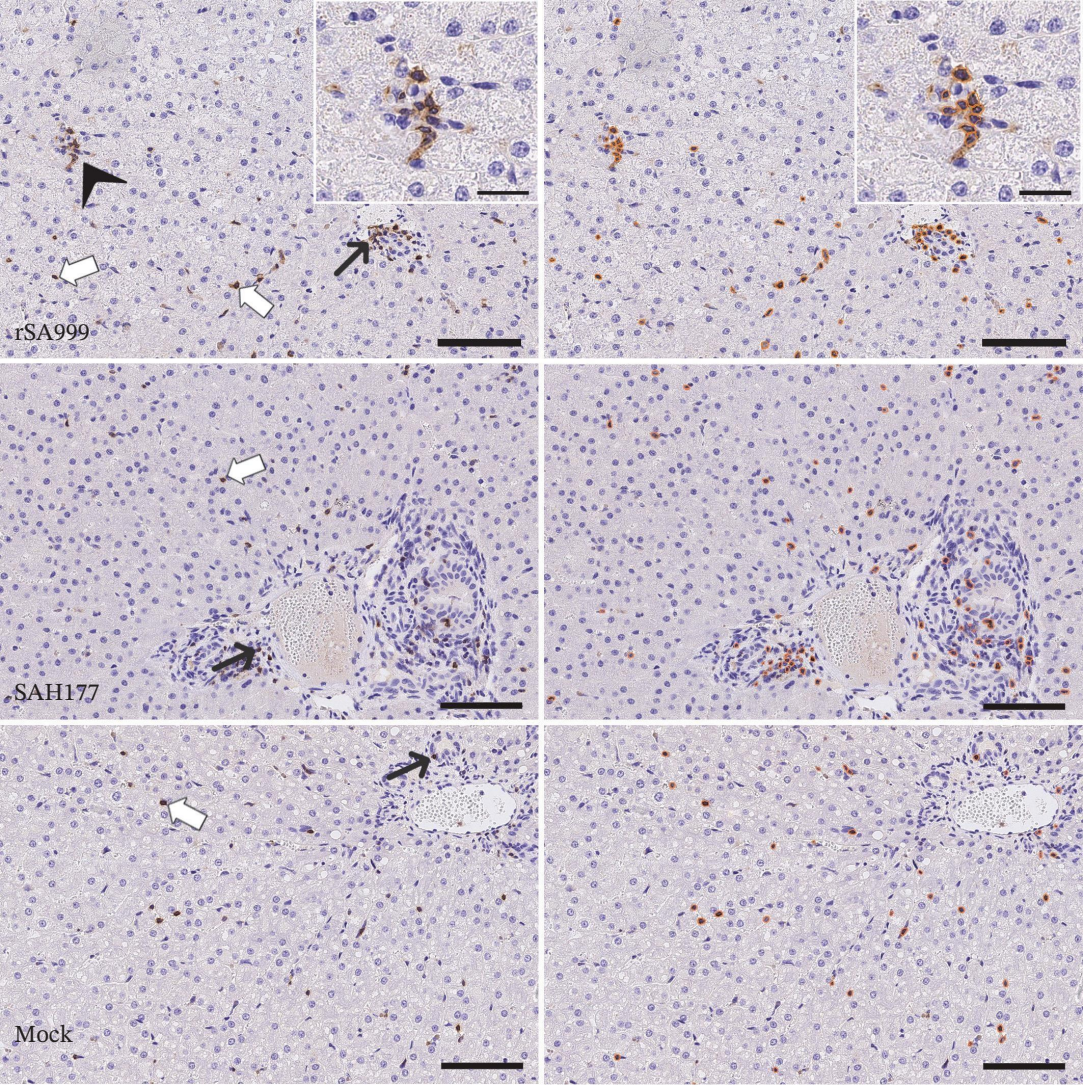
(A)

**Figure:**
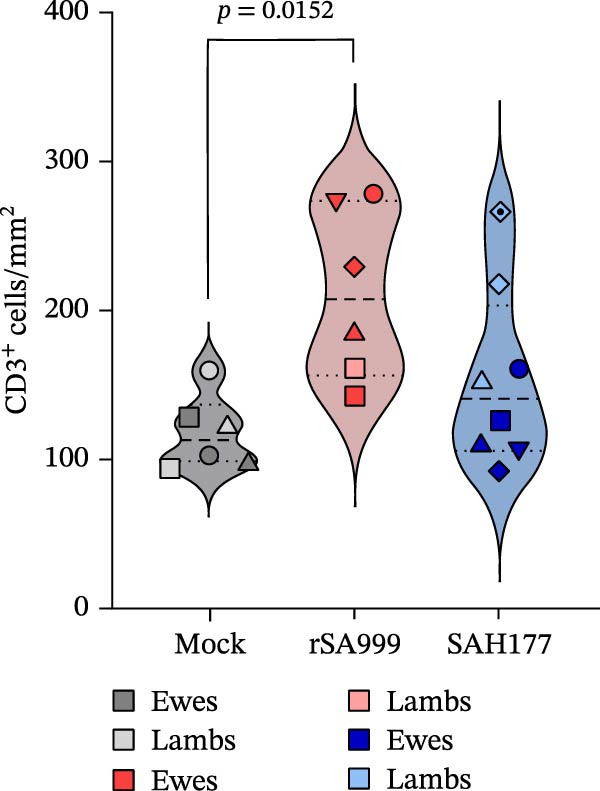
(B)

### 3.3. PAX5

Increased levels of activated B cells in the liver have been demonstrated in studies on hepatitis C virus (HCV) and hepatitis B virus (HBV) [15–17]. B lymphocytes were identified as PAX5^+^ cells and found to have a tenfold lower density than T lymphocytes. Most PAX5^+^ cells were located within the hepatic portal areas in ewes (Figure 2A), often forming follicular‐like aggregates and associated with T cells and a variable degree of bile duct hyperplasia and cholangitis (Figure 2A). These portal changes were not observed in lambs. Only a few individually scattered PAX5^+^ B lymphocytes were seen within the sinusoids of both ewes and lambs. A higher density of PAX5^+^ cell density was found in the rSA999 group (Figure 2A) compared to the SAH177 and mock groups (*p*  = 0.0168 and*p* = 0.0475, respectively; Figure 2A,B. When comparing ewes and lamb regardless of the infection status, ewes had a higher PAX5–positivity cell density than the lambs (*p* = 0.0065).

Figure 2Quantitative digital analysis of PAX5 immunohistochemistry. (A) Depicts the histological liver appearance from three representative lambs: one infected with WSLV strain rSA999 (top), one with WSLV strain SAH177 (middle), and one mock (bottom). Images on the left are unlabeled, whereas corresponding images on the right include orange digital labels outlining PAX5–positive cells are presented on the right. Low numbers of PAX5–positive cells are scattered within the liver parenchyma of all three animals (white arrows). Scale bar: 90 µm. (B) PAX5–positive cell density. Results are presented as number of PAX5–positive cells/mm^2^ of liver parenchyma. Distinct symbols are used to represent different animals within each group as depicted by Zimoch et al. [6]; lambs have the same symbol as their mother; twin lambs from the SAH177 group are distinguished by a dotted symbol.

**Figure:**
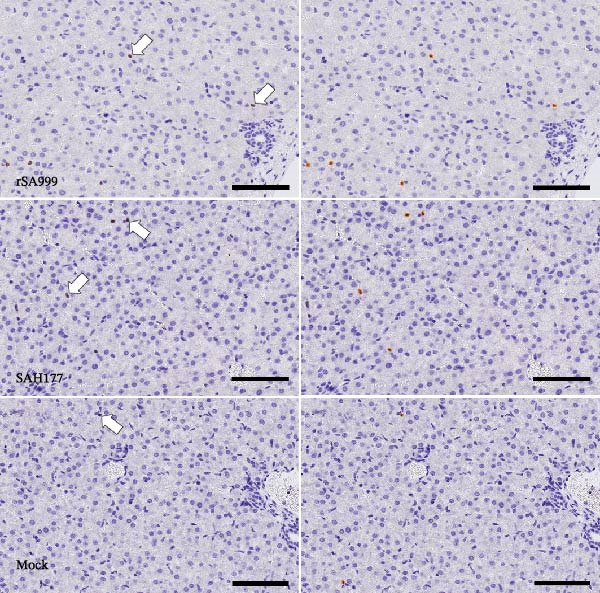
(A)

**Figure:**
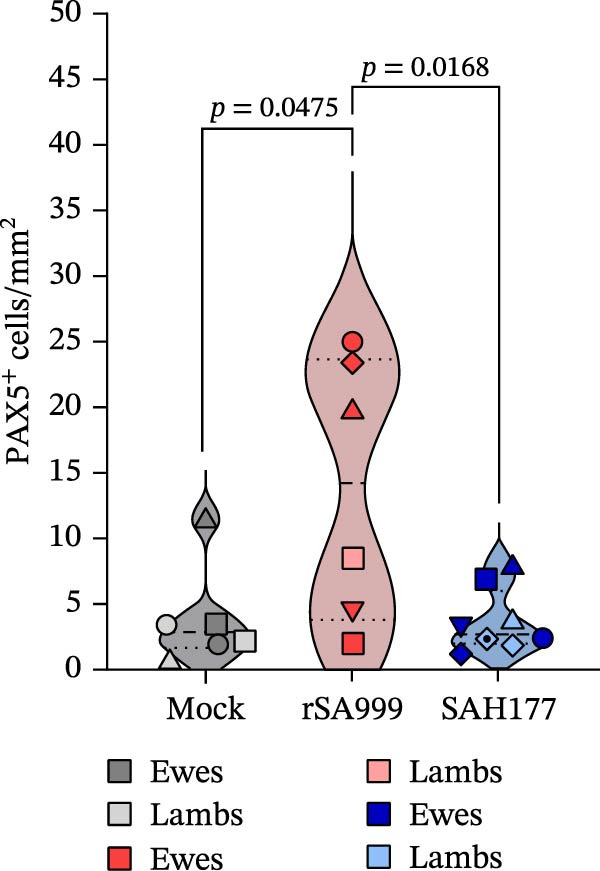
(B)

### 3.4. Iba1

Iba1 labeling detects both the hepatic resident macrophages (Kc) and the monocyte derived macrophages (MDMs) recruited upon infection or liver injury [18, 19]. Iba1 labeling revealed numerous elongated to round cells forming clusters predominantly in the sinusoids and portal tracts (Figure 3A). Livers from WSLV–infected sheep presented higher Iba1^+^ density compared to uninfected animals (Figure 3A). While this increase appeared more pronounced in rSA999–infected sheep (Figure 3A,B, *p* = 0.009) than in the SAH177–infected animals (Figure 3A,B, *p* = 0.0217), differences between infected groups were not statistically significant.

Figure 3Quantitative digital analysis of Iba1 immunohistochemistry. (A) Depicts the histological liver appearance from three representative lambs: one infected with WSLV strain rSA999 (top), one with WSLV strain SAH177 (middle), and one mock (bottom). Images on the left are unlabeled, whereas corresponding images on the right include yellow digital labels outlining Iba1–positive cells presented on the right. Numerous elongated to round Iba1–positive cells are present within the sinusoids (white arrows) and portal tracts (asterisks), being more prominent in the rSA999–infected ewe than in the other two. Scale bar: 90 µm. (B) Iba1 positivity (relative). Results are presented as the percentage of Iba1–positive area in relation to the total examined liver area. Distinct symbols are used to represent different animals within each group as depicted by Zimoch et al. [6]; lambs have the same symbol as their mother; twin lambs from the SAH177 group are distinguished by a dotted symbol.

**Figure:**
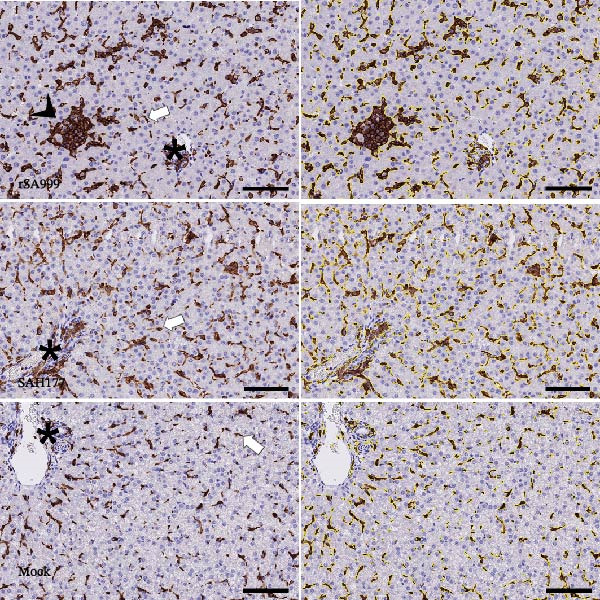
(A)

**Figure:**
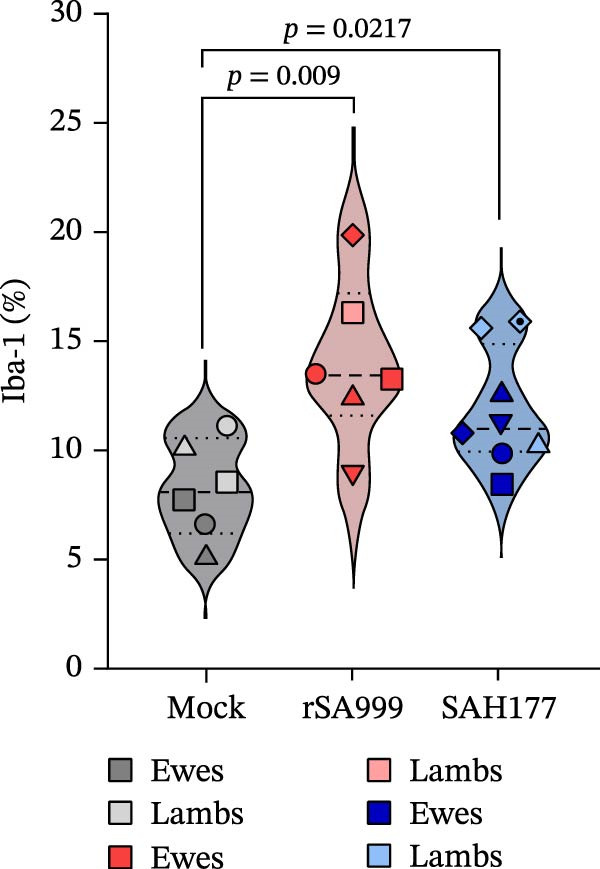
(B)

### 3.5. Ki67 Proliferative Index (PI) in Hepatocytes

The proliferation antigen Ki67 is expressed in the nucleus of cells that are replicating [20], and Arg1 is uniformly expressed in cytoplasm of hepatocytes [21]. The double Ki67 and Arg1 IHC allowed us to calculate the Ki67 PI of the hepatocytes, excluding other cell types present in the liver. WSLV–infected animals showed a higher PI, indicating increased hepatocyte replication than in the control animals (Figure 4A,B, *p* = 0.0033). While there were no significant differences between infected groups, we observed a stronger significance for the rSA999 group than for the SAH177 group compared to the control group (*p* = 0.0087 and *p* = 0.0173, respectively; Figure 4B).

Figure 4Quantitative digital analysis of double arginase 1‐Ki67 immunohistochemistry. (A) Depicts the histological liver appearance from three representative lambs: one infected with WSLV strain rSA999 (top), one with WSLV strain SAH177 (middle), and one mock (bottom). The images show the hepatocytes being diffusely positive for arginase 1 (intracytoplasmic and intranuclear red staining), while Ki67–positive nuclei are stained in brown. Images on the left are unlabeled, while corresponding images on the right include blue digital labels surrounding Ki67–negative hepatocellular nuclei (arginase 1 positive and Ki67 negative cells) and yellow digital labels surrounding Ki67–positive hepatocellular nuclei (arginase 1 positive and Ki67–positive cells). The number of Ki67–positive hepatocytes is higher in the rSA999–infected ewe than in the SH177 and the mock. Scale bar: 90 µm. Inset: Higher magnification of the corresponding image showing detail of the double stain, including Ki67–negative hepatocytes (nuclei with blue labels on the left), Ki67–positive hepatocytes (nuclei with yellow labels on the right), and nonhepatocytic cells (cells negative for arginase 1). Scale bar: 25 µm. (B) Proliferative index (PI) is calculated only in arginase 1–positive cells (hepatocytes). Results are presented as (Ki67–positive nuclei/total nuclei) × 100%. Distinct symbols are used to represent different animals within each group as depicted by Zimoch et al. [6]; lambs have the same symbol as their mother; twin lambs from the SAH177 group are distinguished by a dotted symbol.

**Figure:**
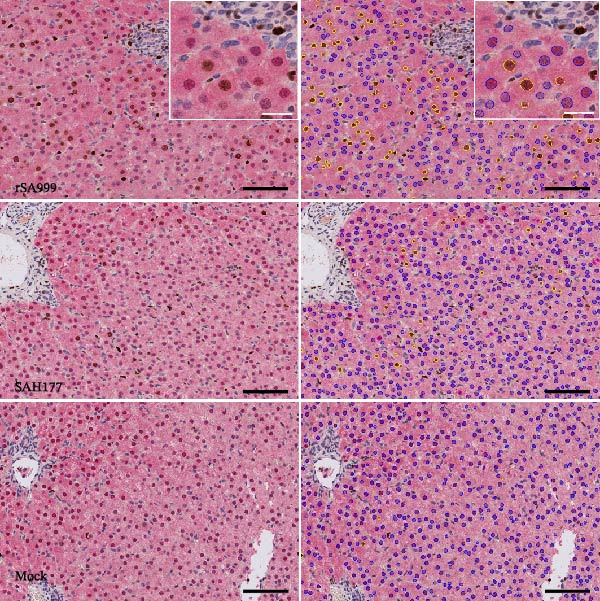
(A)

**Figure:**
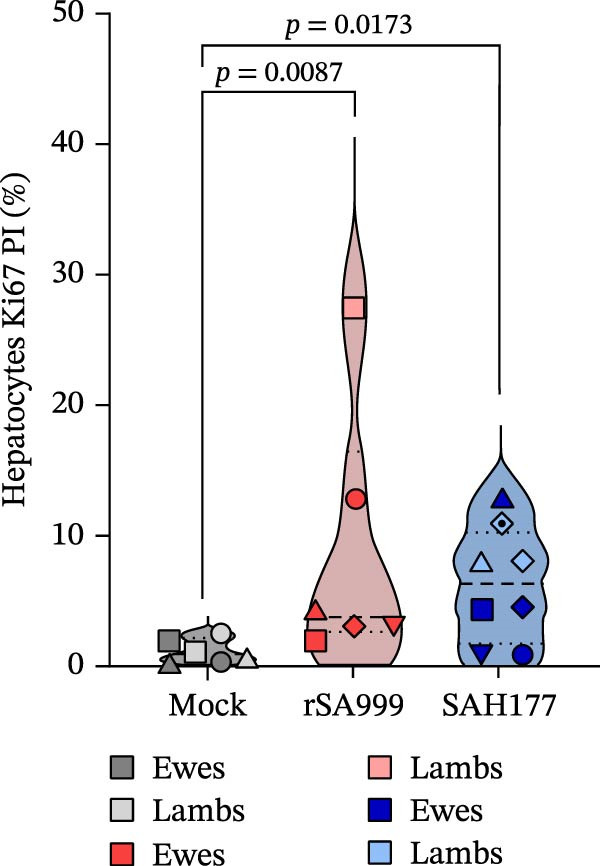
(B)

### 3.6. WSLV NS1 IHC

As previously described, the WSLV NS1 IHC staining pattern was coarsely granular, predominantly in the cytoplasm of hepatocytes and Kc, and possibly also extracellular (Figure 5A) [6]. No WSLV NS1 positivity was observed in the liver from mock animals. However, a certain background staining was present in most of the slides. On this line, the digital analysis of samples from mock animals was all close to 0 and below a threshold value of 0.05% in all of them (Figure 5B). Although not significant, WSLV NS1 IHC staining in the rSA999 group showed a trend to be higher than in the SAH177 group (*p* = 0.075, Figure 5B).

Figure 5Quantitative digital analysis of WSLV immunohistochemistry. (A) Depicts the histological liver appearance from two representative lambs: one infected with WSLV strain rSA999 (top) and one with WSLV strain SAH177 (bottom). Images on the left are unlabeled, whereas corresponding image in the right includes yellow digital labels surrounding IHC WSLV staining. The WSLV IHC positivity is coarsely granular, predominantly within the hepatocytes (arrowheads) and Kupffer cells (black arrow). The staining is more abundant and forms larger aggregates in the rSA999–infected lamb than in the SAH177–infected one. (B) IHC WSLV staining (relative). Results are presented as the percentage of WSLV–positive area in relation to the total examined liver area. Distinct symbols are used to represent different animals within each group as depicted by Zimoch et al. [6]; lambs have the same symbol as their mother; twin lambs from the SAH177 group are distinguished by a dotted symbol.

**Figure:**
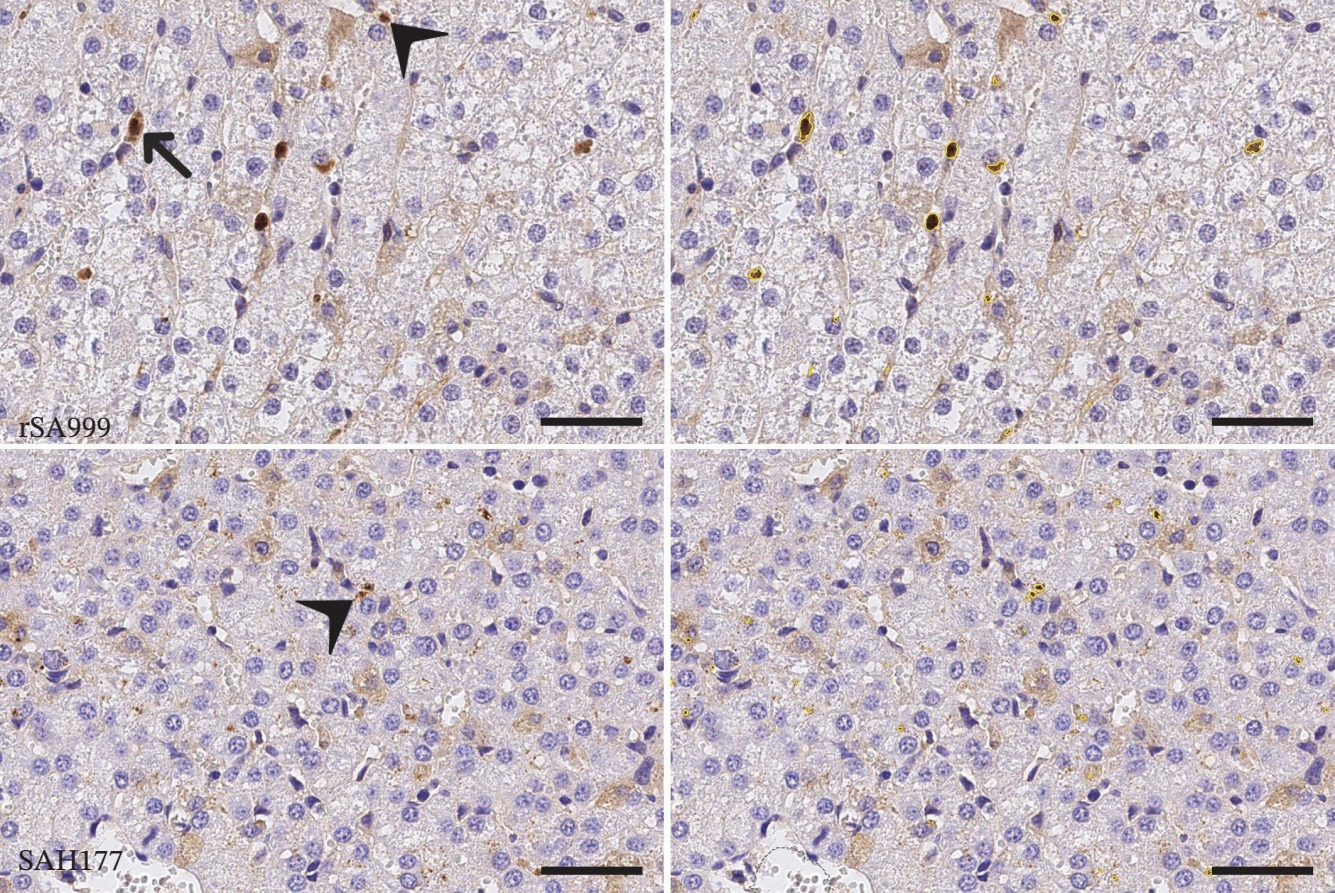
(A)

**Figure:**
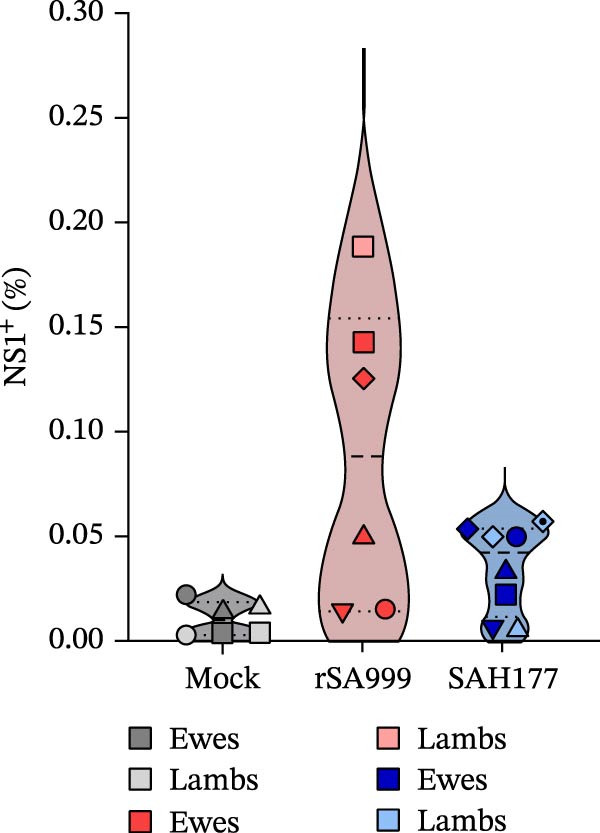
(B)

### 3.7. Correlation Between IHC Markers, WSLV Viral Load, and Serum Markers of Hepatitis

**Figure 6:**
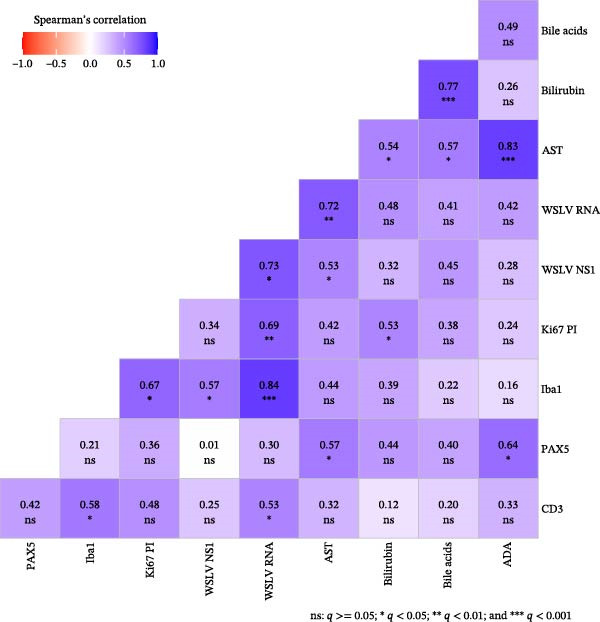
Correlation matrix. Spearman’s correlation values between digitally assessed parameters (IHC for CD3, Iba1, PAX5, Ki67 proliferative index in hepatocytes, and WSLV NS1), qRT‐qPCR results, and serum markers of liver disease (AST, bilirubin, bile acids, and ADA). Blue: positive correlation; red: negative correlation. Ns: *q* > = 0.05;  ^∗^: *q* < 0.05;  ^∗∗^: *q* < 0.01; and  ^∗∗∗^: *q* < 0.001.

**Figure 7:**
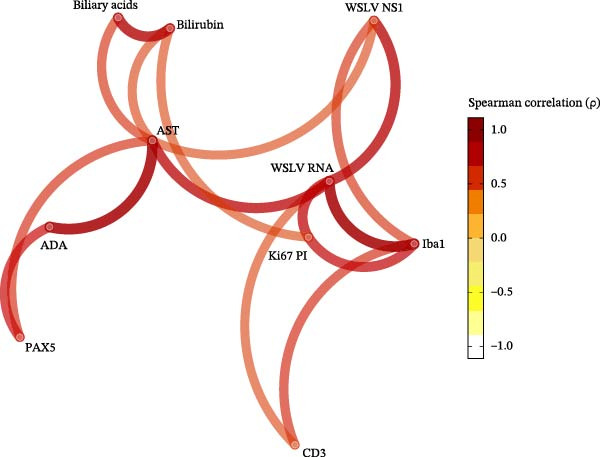
Correlation network analysis. The network displays statistically significant intercorrelations among the assessed parameters, including digitally quantified immunohistochemical markers (CD3, Iba1, PAX5, WSLV NS1, and Ki67 proliferative index in hepatocytes), qRT‐qPCR results, and serum markers of liver disease (AST, bilirubin, bile acids, and ADA). The heatmap scale represents the range of Spearman’s Rho (ρ) values, with dark red indicating the strongest positive correlations and white indicating no correlation.

Finally, we investigated correlations between the digital pathology quantifications presented above with previously published WSLV viral load in liver and serum markers of liver disease from the same animals [6] (Figure 6). All digitally assessed parameters, except PAX5, were positively correlated with WSLV RNA levels, with the strongest correlation being with Iba1 (*p* = 0.84 and *q* < 0.0001), followed by WSLV NS1 IHC (*p* = 0.73, *q* = 0.0013), Ki67 PI (*p* = 0.69, *q* = 0.003), and CD3^+^ cells (*p* = 0.53, *q* = 0.042) (Figure 6). The levels of the liver damage marker AST correlated positively with both WSLV NS1 and PAX5 density, while bilirubin levels were correlated with the proliferation marker Ki67, and ADA levels correlated with PAX 5 staining. The complete correlation data (Spearman’s coefficients, raw *p*‐values, and FDR–adjusted *q*‐values) are provided in Supporting Information file 1. Further, a correlation network analysis displays this study’s relevant and statistically significant positive correlations (Figure 7). This shows the different connections between parameters and helps us better understand the hepato‐pathology caused by WSLV infection in sheep. Clearly, we can distinguish a first cluster of intercorrelations, where it is evident that WSLV RNA levels are strongly linked to high levels of NS1 protein and Iba1–positive cells (including Kc and MDM, natural target for WSLV infection), then in a lower degree to the Ki67 PI and the recruitment of CD3^+^ cells. This group of markers is linked to a cluster that reflects hepatocellular damage defined by AST, bilirubin, and bile acids, which is at the same time linked to inflammation and immune response activation (ADA) with a B cell recruitment (PAX5) component.

## 4. Discussion

By integrating whole‐slide imaging with machine learning–driven quantitative histopathology, digital pathology provides a powerful approach to systematically characterize tissue alterations in infectious diseases [11]. Here, we performed a deep learning based, automated, in‐depth digital analysis of IHC–stained liver tissues from ewes and lambs with mild to moderate WSLV–induced hepatic lesions. This analysis revealed that, at 12 dpi and under the conditions of this experiment, WSLV–infection is associated with increased lymphocytic and macrophage lineage cell populations within the hepatic parenchyma. Among the parameters assessed, the correlation between Iba1 density and WSLV RT‐qPCR was the strongest observed, underscoring the central involvement of histiocytes in WSLV–induced hepatitis. The correlation analysis suggests that the viral replication in hepatocytes (WSLV RNA quantification and NS1 expression) results in hepatocellular damage (AST and bilirubin), which is clearly linked to inflammation (ADA) and an active immune response to the viral infection (ADA, Iba1, CD3, and PAX5). Moreover, the in‐depth digital analysis allowed us to identify group effects, showing a stronger lymphocytic infiltration in the rSA999–infected animals than in the SAH177–infected ones. This finding supports the clinical biochemistry data that rSA999 induces more prominent hepatitis than SAH177 [6]. In addition, this study shows an increased Ki67 PI in the hepatocytes of WSLV–infected animals compared to controls, demonstrating an increased hepatic regeneration in infected animals at the end of the study.

Hepatic macrophages highly contribute to liver immunity and participate in hepatic homeostasis, inflammation, and repair during acute and chronic injury [22]. Iba1 has been used to detect macrophages in the liver, including Kc and infiltrating MDM across species including humans, ruminants, and laboratory animals [23]. In our study, the increased Iba1 positivity observed in WSLV–infected animals suggests macrophage activation, including both Kc and additional infiltrating MDM, as part of the immune response, as described in other viral hepatitis [10, 24]. This activation was more pronounced in lambs, which aligns with the previously reported more severe clinical signs and higher levels of hepatic biochemical markers (including ALT, AST, bile acids, and bilirubin) in lambs than ewes [3, 6]. Indeed, the influx of MDM and their activation is a characteristic feature of the so‐called necroinflammatory phase of hepatitis, the first phase of the wound healing response upon liver injury [22]. In this context, the correlation analysis suggests that the WSLV hepatic infection leads to both higher Iba1 positivity and increased Ki67 PI, thus suggesting a link between Kc and MDM and the hepatic regenerative response. The activation of a regenerative hepatic response in WSLV–infected animals had previously been suggested based on nonquantitative observations of increased number of mitotic figures and hepatocytes with large nuclei [9]. The higher Ki67 PI of hepatocytes in WSLV–infected animals compared to the controls observed in our study confirms these previous qualitative observations.

WSLV RNA quantification, Iba1 positivity, and Ki67 PI were intercorrelated with increased CD3^+^ cells in the liver of infected animals. This finding highlights the relevance of the T lymphocytes in the WSLV–induced hepatitis, which may be triggered by the primary viral target cells in the liver, hepatocytes, and macrophages. On this line, the highest density of CD3^+^ cells observed in WSLV–infected animals indicates a hepatic T cell response, which is known to play an important role in the immune response of numerous hepatotropic viruses, including several orthoflavivirus such as the closely related yellow fever virus [25, 26]. T cells are regarded as a key factor in viral clearance, but they may also be responsible for hepatic damage [14, 25]. The histological association of lymphocytes with necrotic hepatocytes could reflect hepatocellular injury inflicted by cytotoxic T lymphocytes [6, 27]. This may eventually occur together with other cells like natural killer (NK) cells, which are able to recognize and kill infected hepatocytes, a mechanism commonly described in several viral hepatitis, including flaviviral hepatitis such as HCV [14, 28]. Studies performed in livers from patients succumbing to fatal yellow fever showed a strong primarily CD4^+^ but also CD8^+^ T cell infiltrate within the hepatic parenchyma [29]. On the other hand, NKT cells are also CD3^+^ but noncytotoxic and an important source of IFNγ, which is a key cytokine for antiviral defense orchestrating immune communication, activating macrophages, and inhibiting viral replication directly [30]. Performing IHC for CD4^+^, CD8^+^, NK, and NKT cells would further increase our knowledge of the hepatic immunological mechanisms induced by WSLV.

In the current study, PAX5^+^ cell density was 10 folds lower than the CD3^+^ cell density, suggesting that B lymphocytes do not play a major role in the hepatic inflammatory response triggered by WSLV. This is similar to other flaviviral‐induced hepatitis like yellow fever [29]. Nevertheless, PAX5^+^ cell density was higher in rSA999 than in the SAH177 group and controls, suggesting that B cells are part of the hepatic inflammatory response induced by this strain. Studies on HCV and HBV showed elevated levels of activated B cells in the liver [15–17]. Particularly, Zhang et al. [17] showed that B cells are recruited to the liver in response to chemokines such as CXCL9, CXCL10, and CXCL11, which are secreted by monocytes and fibroblasts, and that this migration is associated with the inflammatory response in the liver [17]. Moreover, our data show higher B cell infiltration in the liver of ewes compared to lambs, regardless of the infectious status. This observation could be explained by the mild liver fluke infestation (*Dicrocoelium dendriticum*) present in the ewes from this study [31, 32].

The positive correlation between WSLV NS1 IHC quantification and the WSLV RT‐qPCR RNA results indicates that the WSLV NS1 IHC quantification could be used not only as a qualitative confirmation of WSLV infection [33] but also as reliable quantification method of the hepatic viral load. This approach can complement the quantification of viral genetic material obtained via RT‐qPCR by providing additional information, such as the cellular and tissue distribution of the virus. NS1 antigen detection in other orthoflaviviruses, like dengue virus, was highly specific but with variably reduced sensitivity compared to the RT‐PCR [34]. Similarly, the performed IHC for the NS1 protein of the WSLV showed 100% specificity but a reduced sensitivity compared with the RT‐qPCR. NS1 is a glycoprotein essential in orthoflavivirus replication, modulating the host immune response [34]. The reduced sensitivity of the IHC is likely because NS1 is only expressed when viral RNA is actively being translated, and therefore, NS1 detection corresponds to active or recent viral replication [34, 35].

## 5. Conclusion

This study represents a fully quantitative, in‐depth digital histopathological analysis of WSLV–induced hepatitis using machine learning–driven, pathologist‐led digital image analysis implemented in commercial software. As far as we know, similar analysis in other orthoflavivirus‐induced hepatitis, like yellow fever, is lacking. The performed DL–based WSI analysis provides detailed and objective new insights into the hepatic manifestations of the disease. The standardized quantitative approach enabled discrimination between WSLV–infected and noninfected animals and between rSA999 and SAH177–infected groups presenting with mild to moderate histological lesions. This study confirms the power of digital histology techniques to detect WSLV–induced tissue effects, even more so if pathologic changes are only subtle. Further expanding the list of tissue markers (e.g., for macrophages and lymphocyte subsets) and samples collected at multiple timepoints postinfection would contribute to a deeper understanding of the immunopathogenesis of the disease and the different possible outcomes of infection.

## Funding

This work was principally funded by a grant from the Multidisciplinary Center for Infectious Diseases (MCID), University of Bern (Grant MA_17 to Charaf Benarafa and Obdulio García‐Nicolás). Open access publishing facilitated by Universitat Bern, as part of the Wiley ‐ Universitat Bern agreement via the Consortium Of Swiss Academic Libraries.

## Disclosure

The funders had no role in study design, data collection and analysis, decision to publish, or preparation of the manuscript.

## Conflicts of Interest

The authors declare no conflicts of interest.

## Supporting Information

Additional supporting information can be found online in the Supporting Information section.

## Supporting information


**Supporting Information** Supporting Information file 1 indicates the Spearman’s Rho coefficients, raw *p*‐values, and Benjamini–Hochberg FDR–adjusted *q*‐values of the correlation analysis between WSLV qRT‐qPCR results, digitally assessed parameters, and all hepatic biochemical markers.

## Data Availability

The data that support the findings of this study are available upon request from the corresponding author. The data are not publicly available due to privacy or ethical restrictions.

## References

[1] Diagne M. M. , Faye M. , and Faye O. , et al.Emergence of Virus Among Black Rat and Humans in Eastern Senegal in 2013, One Health-Amsterdam. (2017) 3, 23–28, 10.1016/j.onehlt.2017.02.001, 2-s2.0-85013298005. PMC5454166 28616499

[2] Eibner G. J. , Graff S. L. , and Hieke C. , et al.Genotypic and Phylogeographic Insights Into a Pre-Epidemic Variant of Wesselsbron Virus Detected in Sylvatic from Semuliki Forest, Uganda, Microbiology Spectrum. (2024) 12, no. 12. 10.1128/spectrum.00914-24 PMC11619370 39530699

[3] Nelson A. N. , Ploss A. , Yount J. , and Bailey A. , Emerging Mosquito-Borne Flaviviruses, mBio. (2024) 15, no. 12, 10.1128/mbio.02946-24. PMC11633211 39480108

[4] Weyer J. , Thomas J. , Leman P. A. , Grobbelaar A. A. , Kemp A. , and Paweska J. T. , Human Cases of Wesselsbron Disease, South Africa 2010–2011, Vector-Borne and Zoonotic Diseases. (2013) 13, no. 5, 330–336, 10.1089/vbz.2012.1181, 2-s2.0-84876913012. 23473219

[5] Ishag H. , El Tigani-Asil E. , and Zawde W. , et al.Molecular Detection of Wesselsbron Virus in Dromedary Camels, Borana Zone, Ethiopia, 2024, Emerging Infectious Diseases. (2025) 31, no. 6, 1263–1265. 40439475 10.3201/eid3106.250130 PMC12123917

[6] Zimoch M. , Grau-Roma L. , and Liniger M. , et al.Mosquito-Independent Milk-Associated Transmission of Zoonotic Wesselsbron Virus in Sheep, PLOS Pathogens. (2024) 20, no. 12, 10.1371/journal.ppat.1012751. PMC11658706 39652585

[7] Weiss K. E. , Haig D. , and Alexander R. A. , Wesselsbron Virus - a Virus not Previously Described, Associated with Abortion in Domestic Animals, Onderstepoort Journal of Veterinary Research. (1956) 27, no. 2, 183–195.

[8] Coetzer J. A. and Theodoridis A. , Clinical and Pathological Studies in Adult Sheep and Goats Experimentally Infected with Wesselsbron Disease Virus, The Onderstepoort journal of veterinary research. (1982) 49, no. 1, 19–22. 6289220

[9] Coetzer J. A. , Theodoridis A. , and Van Heerden A. , Wesselsbron Disease, Pathological, Haematological and Clinical Studies in Natural Cases and Experimentally Infected New-Born Lambs, The Onderstepoort journal of veterinary research. (1978) 45, no. 2, 93–106. 714395

[10] Flores Molina M. , Abdelnabi M. N. , and Mazouz S. , et al.Distinct Spatial Distribution and Roles of Kupffer Cells and Monocyte-Derived Macrophages in Mouse Acute Liver Injury, Frontiers in Immunology. (2022) 13, 10.3389/fimmu.2022.994480, 994480. 36248843 PMC9562324

[11] Fatima G. , Alhmadi H. , and Ali Mahdi A. , et al.Transforming Diagnostics: A Comprehensive Review of Advances in Digital Pathology, Cureus. (2024) 16, no. 10, 10.7759/cureus.71890. PMC11573928 39564069

[12] Dietert K. , Nouailles G. , and Gutbier B. , et al.Digital Image Analyses on Whole-Lung Slides in Mouse Models of Acute Pneumonia, American Journal of Respiratory Cell and Molecular Biology. (2018) 58, no. 4, 440–448, 10.1165/rcmb.2017-0337MA, 2-s2.0-85045202120. 29361238

[13] Wanninger T. G. , Saldarriaga O. A. , and Arroyave E. , et al.Hepatic and Pulmonary Macrophage Activity in a Mucosal Challenge Model of Ebola Virus Disease, Frontiers in Immunology. (2024) 15, 10.3389/fimmu.2024.1439971, 1439971. 39635525 PMC11615675

[14] Lopez-Scarim J. , Nambiar S. M. , and Billerbeck E. , Studying T Cell Responses to Hepatotropic Viruses in the Liver Microenvironment, Vaccines. (2023) 11, no. 3, 10.3390/vaccines11030681, 681. 36992265 PMC10056334

[15] Patel A. M. , Liu Y. S. , and Davies S. P. , et al.The Role of B Cells in Adult and Paediatric Liver Injury, Frontiers in Immunology. (2021) 12, 10.3389/fimmu.2021.729143. PMC8495195 34630404

[16] van Buuren N. , Ramirez R. , and Turner S. , et al.Characterization of the Liver Immune Microenvironment in Liver Biopsies from Patients With Chronic HBV Infection, JHEP Reports. (2022) 4, no. 1, 10.1016/j.jhepr.2021.100388, 100388. 34950863 PMC8671126

[17] Zhang J. W. , Lai R. M. , and Wang L. F. , et al.Varied Immune Responses of HBV-Specific B Cells in Patients Undergoing Pegylated Interferon-Alpha Treatment for Chronic Hepatitis B, Journal of Hepatology. (2024) 81, no. 6, 960–970, 10.1016/j.jhep.2024.06.033. 38992769

[18] Guselnikova V. V. , Razenkova V. A. , and Kirik O. V. , et al.Detection of Tissue Macrophages in Different Organs Using Antibodies to the Microglial Marker Iba-1, Doklady Biochemistry and Biophysics. (2024) 519, no. 1, 506–511, 10.1134/S160767292470114X. 39283555

[19] Sendera R. , Weiss Y. , and Navon Y. , et al.The Total Mass, Number, and Distribution of Immune Cells in the Human Body, Proceedings of the National Academy of Sciences. (2023) 120, no. 44, 10.1073/pnas.2308511120. PMC10623016 37871201

[20] Sobecki M. , Mrouj K. , and Camasses A. , et al.The Cell Proliferation Antigen Ki-67 Organises Heterochromatin, eLife. (2016) 5, 10.7554/eLife.13722, 2-s2.0-84964270637. PMC4841783 26949251

[21] Larson B. K. , Dhall D. , and Guindi M. , Arginase-1 Is More Specific Than Hepatocyte Paraffin 1 for Differentiating Hepatocellular Carcinomas With Cytoplasmic Clearing from Nonhepatocellular Clear Cell Tumors in Liver Biopsies, Applied Immunohistochemistry & Molecular Morphology. (2024) 32, no. 1, 37–43, 10.1097/PAI.0000000000001169. 37859468

[22] Hassan G. S. , Flores Molina M. , and Shoukry N. H. , The Multifaceted Role of Macrophages during Acute Liver Injury, Frontiers in Immunology. (2023) 14, 10.3389/fimmu.2023.1237042, 1237042. 37736102 PMC10510203

[23] Donovan K. M. , Leidinger M. R. , and McQuillen L. P. , et al.Allograft Inflammatory Factor 1 as an Immunohistochemical Marker for Macrophages in Multiple Tissues and Laboratory Animal Species, Comparative Medicine. (2018) 68, no. 5, 341–348, 10.30802/AALAS-CM-18-000017, 2-s2.0-85055627198. 30227902 PMC6200031

[24] Tacke F. and Zimmermann H. W. , Macrophage Heterogeneity in Liver Injury and Fibrosis, Journal of Hepatology. (2014) 60, no. 5, 1090–1096, 10.1016/j.jhep.2013.12.025, 2-s2.0-84898789996. 24412603

[25] Kuhn R. J. , Barrett A. D. T. , and Desilva A. M. , et al.A Prototype-Pathogen Approach for the Development of Flavivirus Countermeasures, The Journal of Infectious Diseases. (2023) 228, no. 6, S398–S413, 10.1093/infdis/jiad193. 37849402 PMC10582523

[26] Watson A. M. and Klimstra W. B. , T Cell-Mediated Immunity towards Yellow Fever Virus and Useful Animal Models, Viruses. (2017) 9, no. 4, 10.3390/v9040077, 2-s2.0-85017656955, 77. 28398253 PMC5408683

[27] Le Roux J. M. W. , The Histopathology of Wesselsbron Disease in Sheep, Onderstepoort Journal of Veterinary Research. (1959) 28, no. 2, 237–243.

[28] Misumi I. , Mitchell J. E. , Lund M. M. , Cullen J. M. , Lemon S. M. , and Whitmire J. K. , T Cells Protect against Hepatitis A Virus Infection and Limit Infection-Induced Liver Injury, Journal of Hepatology. (2021) 75, no. 6, 1323–1334, 10.1016/j.jhep.2021.07.019. 34331968 PMC8604763

[29] Quaresma J. A. S. , Barros V. L. R. S. , and Pagliari C. , et al.Hepatocyte Lesions and Cellular Immune Response in Yellow Fever Infection, Transactions of the Royal Society of Tropical Medicine and Hygiene. (2007) 101, no. 2, 161–168, 10.1016/j.trstmh.2006.02.019, 2-s2.0-33751543068. 16872652

[30] Schroder K. , Hertzog P. J. , Ravasi T. , and Hume D. A. , Interferon-γ: An Overview of Signals, Mechanisms and Functions, Journal of Leukocyte Biology. (2004) 75, no. 2, 163–189, 10.1189/jlb.0603252, 2-s2.0-0842266786. 14525967

[31] Ferreras-Estrada M. C. , Campo R. , González-Lanza C. , Pérez V. , García-Marín J. F. , and Manga-González M. Y. , Immunohistochemical Study of the Local Immune Response in Lambs Experimentally Infected with *Dicrocoelium dendriticum* (Digenea), Parasitology Research. (2007) 101, no. 3, 547–555, 10.1007/s00436-007-0511-1, 2-s2.0-34547117582. 17393185

[32] Pérez J. , de las Mulas J. M. , De Lara F. C. M. , Gutierrez-Palomino P. N. , Becerra-Martel C. , and Martínez-Moreno A. , Immunohistochemical Study of the Local Immune Response to in primarily and secondarily infected goats, Veterinary immunology and immunopathology. (1998) 64, no. 4, 337–348. 9764726 10.1016/s0165-2427(98)00144-5

[33] van der Lugt J. J. , Coetzer J. A. , Smit M. M. , and Cilliers C. , The Diagnosis of Wesselsbron Disease in a New-Born Lamb by Immunohistochemical Staining of Viral Antigen, Onderstepoort Journal of Veterinary Research. (1995) 62, no. 2, 143–146. 8600439

[34] Fisher R. , Lustig Y. , Sklan E. H. , and Schwartz E. , The Role of NS1 Protein in the Diagnosis of Flavivirus Infections, Viruses. (2023) 15, no. 2, 10.3390/v15020572, 572. 36851784 PMC9963814

[35] Macknezie J. M. , Jones M. K. , and Young Y. R. , Immunolocalization of the Dengue Virus Nonstructural Glycoprotein NS1 Suggests a Role in Viral RNA Replication, Virology. (1996) 220, no. 1, 232–240, 10.1006/viro.1996.0307, 2-s2.0-0029941322. 8659120

